# Beyond the checklist: understanding rural health vulnerability in a South African context

**DOI:** 10.3402/gha.v9.33272

**Published:** 2016-11-30

**Authors:** Richard Vergunst, Leslie Swartz, Gubela Mji, Janis Kritzinger, Stine Hellum Braathen

**Affiliations:** 1 Department of Psychology, University of Stellenbosch, Stellenbosch, South Africa; 2 Centre for Rehabilitation Studies, University of Stellenbosch, Stellenbosch, South Africa; 3 SINTEF Technology and Society, Department of Health, PB 124 Blindern, 0314 Oslo, Norway

**Keywords:** Rural, Vulnerability, Health, South Africa, Checklist

## Abstract

**Background:**

Vulnerability in the past has sometimes been measured and understood in terms of checklists or common understanding. It is argued here that vulnerability is a more complex issue than this. Although checklists of vulnerable groups are important, they do not capture the essence and dynamics of vulnerability.

**Objective:**

The case of rural health vulnerability in South Africa is discussed to show that classifying people into vulnerable groups does not portray the complexity and intricacies of what it means to have vulnerability. We also wish to show that there are different kinds of vulnerabilities, and the difference between access vulnerability and illness vulnerability is highlighted.

**Methods:**

As part of a larger study, this case study is presented to show how vulnerability in a poor rural community in South Africa has to be understood in a contextual and dynamic manner as opposed to a static manner.

**Results:**

Family and social dynamics can influence health. For example, fractured families were seen as a vulnerable issue within the community, while being a person with a disability can lead to isolation and callous attitudes towards them. It is these family and social dynamics that lead proximally to vulnerability to ill health.

**Conclusions:**

A contextual approach can assist in giving a more layered understanding of vulnerability than a checklist approach can do. Interventions to change health cannot be addressed simply by medical means. Social conditions need to be changed, and part of changing social conditions is the process of assisting those who are isolated or experience themselves as vulnerable to reconnect with others in the community. Poverty leads to social exclusion; social and family inclusion may be key to well-being.

## Introduction

An increasing awareness of the problems and suffering posed by the human condition of vulnerability calls for reflection on an ethos of vulnerability (1). We need to have a relook at what the term ‘vulnerability’ means in research. Despite the fact that the term is frequently covered in social science research, there are problems regarding the application of this concept in terms of analysis and measurement (2). Defining and analysing vulnerability is a difficult task. There is no clear understanding, interpretation and application of vulnerability in the academic literature (3). Yet the concept of vulnerability is important because of its implications for health. A review of health literature reveals that the term ‘vulnerability’ is commonly used, yet there is no comprehensive consensus on the precise definition. Indeed, even where researchers are at great pains to engage with the question of the definition of other terms related to health, the term ‘vulnerability’ may be presented without a definition and as seemingly self-evident (4). The term may be left open to individual interpretation, and it may be difficult consequently to apply the term to practice (5). This concept needs to be clarified so that one can use it more effectively (6). This is especially important when the term ‘vulnerability’ is used, not in relation to risk for a particular discrete event such as a trauma or the onset of a health condition, but when ‘vulnerability’ as a concept is used in the context of long-term health conditions (7).

When issues of health are considered in the context of vulnerability, the term ‘vulnerability’ assumes a number of specific meanings. The two major classes of meaning involve vulnerability to illness itself – it is well established that poor people are more vulnerable to a range of illnesses – and vulnerability to poor health care or to lack of access to health care – which people may experience. These two forms of vulnerability, which may be termed ‘illness vulnerability’ and ‘access vulnerability’, may be intertwined, but they should not be conflated.

Despite the fact that it is pertinent to address the health needs of vulnerable groups in low-income countries, there are still obstacles and challenges in addressing these needs for these differing groups. Vulnerable groups are ‘social groups who experience limited resources and consequent high relative risk for morbidity and premature mortality’ (8, p. 69), and this may include women, children, the aged, ethnic minorities, displaced people, people suffering from some illnesses and people with disabilities.

It is possible, and useful, to list vulnerability groups which may affect health. A tool, referred to as EquiFrame, has been developed to evaluate and promote the inclusion of vulnerable groups and core concepts of human rights in health policy documents (6, 9). EquiFrame lists a number of vulnerability groups which may affect access to health care (Table 1).

**Table 1:** EquiFrame vulnerable groups definitions

Number	Vulnerable group	Attributes or definitions
1	Limited resources	Poor people or people living in poverty
2	Increased relative risk for morbidity	People with one of the top 10 illnesses identified by WHO as occurring within the relevant country
3	Mother-child mortality	Factors affecting maternal health and child health (0–5 years)
4	Female-headed household	Households headed by a woman
5	Children with special needs	Children marginalised by special contexts, such as orphans or street children
6	Aged	Referring to older age
7	Youth	Referring to younger age without identifying gender
8	Ethnic minorities	Non-majority groups in terms of culture, race or ethnic identity
9	Displaced populations	People who have been displaced from their previous residence because of civil unrest or unsustainable livelihoods
10	Living away from services	People living far from health services, either due to travel time or due to distance
11	Suffering from chronic illness	People who have an illness requiring continuous care
12	Disabled	Persons with disabilities, including physical, sensory, intellectual or mental health conditions, and including synonyms of ‘disability’

It would be possible to go through each of these groups and spell out how each of them may affect illness vulnerability and/or access vulnerability. Although this approach is useful in drawing attention to factors that must be taken into account in understanding health vulnerabilities, a broad instrument cannot tease out the dynamics of vulnerability in particular contexts (nor is it designed to do so). As Ten Have (3) mentions, categorising groups and populations as vulnerable lacks subtlety. Every context has its own history, geography and set of social dynamics. We begin with an overview of historical factors affecting health and health care in rural South Africa, because it is against this backdrop that contemporary issues and challenges are experienced.

### The context of health care delivery in South Africa

Of the total population in South Africa, 52% live in rural areas where 75% of poor South Africans live (10). The political situation over the last 50 years in South Africa has influenced the rural practice in the country (11). Rural health in South Africa has parallels with the health of people living in poverty, and in the deliberately underdeveloped areas of the country, inhabited largely by Black community members. Since the beginning of democracy back in 1994, there have been plans for sweeping changes to the health care system, and the priority principle of health care plans was that of equity (12, 13). Equity has direct implications for rural health care and practice in South Africa. The quality of rural health care services can be seen as a barometer of success of the broader social reforms undertaken by the government.

South African society is undergoing change, and this is shown in its morbidity, mortality and disability profiles (11). The health status of rural people in South Africa is comparable with that of people in many other developing nations across the world. The diseases of poverty including chronic disability are common. Access to health care for rural people is difficult: the high cost of transport and the large distances involved lead to late presentations of disease, particularly in rural areas. This is further complicated by traditional beliefs regarding illnesses: unregulated traditional healers of various levels of experience and skill make their services available to a somewhat fearful and tradition-bound public in rural areas.

The public health care system in South Africa operates in terms of layers, with referral paths from primary health care facilities to secondary hospitals and, ultimately, to specialist tertiary facilities. In rural areas, this translates into a system of rural hospitals and clinics. These were primarily built and operated as mission hospitals until the 1970s when most of them were taken over by the apartheid government in an effort to centralise planning. The platform for the new National Health System was based on these hospitals, and they were there to develop a district-based health system away from centralisation. The infrastructure and facilities available in rural hospitals are relatively good, although some services had limitations. Most rural hospitals, such as Madwaleni Hospital in the rural Eastern Cape, offer a comprehensive service and include generalist doctors who are largely foreign-qualified.

When it comes to vulnerability and health access in South Africa, a useful yardstick would be to assess the quality of care offered to an elderly woman with disability living in a rural area in South Africa as the issues raised by care for this person exemplify complex challenges (10). This study discusses a number of cases in a rural area in South Africa to explore vulnerability issues. There have been no other studies found in South Africa that have addressed this particular issue in this way.

### Research question

The research question for this particular study is: how is vulnerability experienced and understood in Madwaleni?

### Research objective

The objective of this study is to show how a contextual approach can assist in giving a more layered understanding of vulnerability than a checklist approach can do.

## Method

We used a case study approach to demonstrate the complexity of issues in determining questions of vulnerability in relation to health care in rural South Africa. We drew on observations and discussions during a 3-year engagement with health system issues in Madwaleni (described below). The engagement formed part of our work on a larger project entitled ‘Enabling Universal and Equitable Access to Health Care for Vulnerable People in Resource Poor Settings in Africa’ (EquitAble). Substantive findings from that project have been reported elsewhere (14, 15).

According to Yin (16), the value of a case study is that it allows for a holistic understanding of complex issues which cannot be easily understood out of context. Both qualitative and quantitative data may be collected. Our approach to the material follows Yin's (16) guidelines, and in our method we also take account of the approach to organisational ethnography outlined by Smith (17). Case studies and ethnographies can be used to describe local situations, and they also have a role in allowing researchers’ insights into theoretical issues and questions (18). Our intention in this article is not primarily to describe a local situation, but instead to draw on 3 years of fieldwork in a rural South African context to develop a theoretical discussion of vulnerability as a conceptual issue. We use a qualitative approach to do this.

Various health care workers and community members of the Madwaleni area were interviewed over a 3-year period as part of the larger EquitAble study. Purposive sampling was used. Six cases – those of three health care workers and three community members – formed part of the sample for this study. Each participant was interviewed once. Data were recorded, transcribed and analysed using the ATLAS TI program.

Ethical clearance was obtained from Stellenbosch University (Ethics Reference No: N09/10/270).

### Vulnerability: a case study in rural South Africa

Having the theoretical background from EquiFrame of how to ascertain vulnerable groups and how to define and measure vulnerability, we now investigate these notions on a practical level.

### Study setting

We will take the case study of Madwaleni – a deeply impoverished rural community in South Africa – with a population of about 120,000 people. Madwaleni is on the Wild Coast in South Africa's Eastern Cape Province (formerly Transkei) situated 30 km from Elliotdale, 100 km from Mthatha and 220 km up the coast from East London. The area is characterised by rugged hills, rivers, forests, unpaved gravel roads, free running animals and grass-thatched huts scattered sporadically over the hills. There is a scarcity of sewage systems, running water and electricity to the general Madwaleni community, and these are limited to the hospital and the local hotels. There are high levels of unemployment within the 20 villages of Madwaleni.

The Madwaleni Hospital is situated in the rolling hills of the Elliotdale District under Mbhashe Local Service Area. The area is also served by eight clinics: Hobeni, Nkanya, Bomvana, Molitafa, Soga, Xhora, Mqhele and Mkhatazo. There are two major rivers (Mbashe and Xora) and several other tributaries and streams. The clinics Madwaleni, Bomvana, Hobeni, Nkanya, Molitafa and Soga are in between the two major rivers, while Xhora and Mqhele are on the outer side of the Xora river.

The population of Madwaleni are Xhosa-speaking, from the Amabovane clan. The hospital staff comprises doctors and allied professionals who are primarily Caucasian, as well as sisters and nurses who are Amabovane and who share linguistic and cultural backgrounds with the patient population. By definition, Madwaleni is a vulnerable community in that they experience limited resources with high risk for morbidity and premature mortality although little exists in the literature regarding the role of vulnerability to rural populations (8). There are also high perceptions of vulnerability in the community – perceived vulnerable groups by the community include children, AIDS orphans, uneducated people, youth, elderly people, people with disabilities including the mentally ill, women, HIV-positive persons, substance users and poor people. The degree of vulnerability is greatly affected by the perception of the individual (19).

## Results

### Views of health workers

The community has been perceived by high-ranking medical officials from the only hospital in the community as a community with ‘fractured families’ and ‘unstable homes’– these are the terms used by our informants and which could be added to a list of vulnerability factors, but which in fact reflect core processes of the vulnerability of rural impoverishment. As a high-ranking health care worker states:

I think the same: I think those are the two main groups. There are individuals who are certainly vulnerable in this community to abuse, but also vulnerable to a lack of opportunity in the sense of the education levels and the community being quite poor. I think the fractured families here affect both women and children because a lot of the men being elsewhere.

and

Vulnerable groups are children with unstable homes.

It is these ‘fractured families’ and ‘unstable homes’ that make the community particularly vulnerable – consequent on a lack of material resources, the resources of reliable family and community ties, and, indeed, of the predictability of life itself.

### Views of people who are regarded as vulnerable

Some people with a disability (listed as a vulnerable group) experience no vulnerability in the community, while others feel very vulnerable. According to a woman with disability in Madwaleni, the community has ‘accepted’ disability and hence has a ‘positive attitude’ towards disability:

Disabled people themselves have taught communities to actually have a positive attitude towards disabled people, because they themselves have actually continued to help that type of attitude – that type of positive attitude towards themselves meant that communities looked at them positively too.

She says that she feels that the government has made a huge impact by changing the perception of society towards people with disabilities, and that has made the community change its attitude. She feels that the government has achieved this by being proactive in integrating people with disabilities into the job market. She also believes that her religion has assisted and supported her, giving her a certain self-centredness.

According to a nursing sister in Madwaleni, there is no vulnerability experienced by people with disabilities in her part of the community because their headman loves people with disabilities:

like I'm saying, because of our headman *loves* the disabled ones. So there is no one who discriminate against those in the community.

There is no discrimination towards people with disabilities in the community as they have a role model who helps to change attitudes and negative stereotypes:

So to have a role model (a leader) who actually acts in a certain way, can also help to take away attitudes and change attitudes.

However, there are people with disabilities in Madwaleni who feel vulnerable – a mother with a child who has a disability states that they really feel quite isolated because, generally, the community tends to distance itself from them, and they are seen as not mentally stable. This is actually quite painful for them. She observes how the community generally has a ‘callous attitude’ and can say ‘callous things’ towards them. When she moves around in the community, she meets all this ‘antagonism’:

They really feel quite isolated because generally, the community tends to distance itself from them, and they are being seen as really not mentally stable. Hence, the community tends to distance itself from them. She is also just saying that they are seen as not stable, and that is actually quite painful. She says for her, she just wants to affirm the perceptions that they have, because she observes how the community just generally has a callous attitude towards them, and they can say whatever they want to say in whatever manner they want to say it. She is always staying in her home. But people come from outside and can just come in and say callous things. For her, she moves around a lot, so she meets all this antagonism when she actually moves around in the community.

Another woman with disability states that people with disabilities are vulnerable and ‘cannot defend for themselves’ in their community:

They know that you as a disabled person, you can't defend yourself. And if they come and open your house to take something, it's difficult because you can't actually fight back.

## Discussion

Not all persons in Madwaleni are vulnerable – it is their particular *context* that they find themselves in that makes them vulnerable or not. As Allotey et al. (20) state, vulnerability is dependent on context, including social and cultural systems and political and economic trends. This is supported in an article by Zarowsky et al. (21), in which it is argued that the ‘real work – at both intellectual and policy/political levels – lies in understanding and responding to the dynamics, meanings and power relations underlying actual instances and processes of vulnerability and harm’ (p. 5).

The differing understandings and experiences of vulnerability amongst people in Madwaleni, who ostensibly share the same vulnerability characteristics, demonstrate both the strengths of having a ‘list’ of vulnerability factors and the weaknesses of this approach. As said earlier, having a list allows us to group people together for analytic purposes, but the listing of factors cannot answer the core questions of community, family and personal dynamics that interact to create an experience of vulnerability. What makes one person with disabilities feel vulnerable in the community, while another person with disabilities does not? What is the context of the person that makes him/her vulnerable or not despite being listed as vulnerable? Are there interplaying issues that make vulnerability a more complex phenomenon – more than just the question of belonging to a group or not? Our data demonstrate that these questions are more complex than they appear.

While vulnerable groups are almost always identified, they are often presented as static categories and not linked to a discussion of particular processes or circumstances that lead to labelling them as ‘vulnerable’ (22). Limits and boundaries are created that tend to become fixed and static when attempting to define groups as vulnerable, and this often leads to a focus on quantitative measurement rather than qualitative understanding (21). It is not enough to label groups as vulnerable; we also need to understand the processes which make them vulnerable and who becomes vulnerable because of these processes. Attention needs to be given to the limitations of static approaches (21). For this reason, we do not believe that a model of vulnerabilities is the most helpful approach here – what is required is an understanding of the fluid complexities of vulnerability.

At the core of the question of vulnerability in Madwaleni is the common context of rural poverty, which affects all who live there. But in order to understand vulnerability, it is important to come to grips with processes that are associated with poverty, and these processes affect people in different ways. Vulnerability has its roots in social and economic conditions (23). Working and living conditions as well as social relationships indeed play a role in vulnerability in Madwaleni. Men and women have been forced to leave the community and seek work and income in faraway cities, breaking up the community and family structures for long periods of time. To list ‘women-headed households’ or ‘child-headed households’ as a further vulnerability factor in a list of factors is correct, but this listing may obscure the relationship between the underlying poverty and its effects on how people live their lives. This context of fractured families, to use our participants’ term – and not just poverty as narrowly understood as a lack of material resources – also makes the community potentially more vulnerable to illnesses. For example, family breakdown for economic reasons increases vulnerability to HIV, as men and women come back from cities infected with HIV and they in turn infect their wives or husbands (24). This often also results in them having HIV-infected children. HIV has also left many orphaned children in the area vulnerable due to their physical vulnerability. There is also a cyclical generational component to vulnerability. Due to the lack of adult input, children may make uninformed decisions about health behaviours, or they may lack assertiveness, which may in turn lead to unwanted pregnancies and possible further HIV infection.

It is not only physical health but also social and mental health in the community that are negatively affected by this context. The context of ‘fractured families’ may result in a high prevalence of substance use in the community, often resulting in abuse of women as well as the experience of mental illnesses by some individuals (25). According to the community leaders, substance use is particularly prominent in the month of December when men and women come back into the community from their city employments. At this time, alcohol abuse is more frequent, bringing more vulnerability to the community. As a result, the people abusing substances are also more vulnerable to health issues.

The case of Madwaleni demonstrates how family and social dynamics can influence health. It is these family and social dynamics that lead proximally to vulnerability to ill health. Though poverty is ubiquitous, family and social exclusion (consequent on poverty) occasion vulnerability – not all persons with limited resources experience vulnerability. In fact, certain Madwaleni community members did not perceive themselves as having limited resources, rather they saw themselves as quite well off in terms of land and stock. However, they were still vulnerable when it came to fractured families and the health consequences of this.

### Strengths and limitations of the study

A strength of this study is that it was an in-depth investigation using a case study approach to understand the unique complexities of assessing and measuring vulnerability. The limitations of the study are that it only looked at one rural area within South Africa and that generalisations to other areas need to be made with caution. Furthermore, only self-reported measures were used, which may introduce bias. This study represents a start at looking at vulnerability in the way we have done.

## Conclusion

According to Aday (26), ‘both the origins and remedies of vulnerability are rooted in the bonds of human communities’ (p. 1). We have noted that the Madwaleni community have what have been termed ‘fractured families’, ‘unstable homes’ and hierarchical systems. These dynamics or systems have weakened social bonds and thus have made the community more vulnerable to illnesses such as HIV and social challenges such as substance use and abuse. As Flaskerud and Winslow (8) state, vulnerability to poor health outcomes is a possible result of a lack of social connectedness and social status. Interventions to change health in Madwaleni cannot be addressed simply by medical means. Social conditions need to be changed, and an aspect of changing social conditions is the process of assisting those who are isolated or experience themselves as vulnerable to reconnect with others in the community. Poverty leads to social exclusion; social and family inclusion may be key to well-being. As Ten Have (3, p. 406) has concluded, as a ‘fundamental expression of the human condition, vulnerability can only be properly addressed if the social dimension of human existence is taken seriously’. A recommendation, therefore, is to contextualise vulnerability and investigate its social dimensions before sometimes putting them down to a checklist.

## Paper context

Vulnerability in the past has sometimes been measured and understood in terms of checklists or common understanding. It is argued here that vulnerability is a more complex issue than this. Although checklists of vulnerable groups are important, they do not capture the essence and dynamics of vulnerability. The aim of the paper was to show how a contextual approach can assist in giving a more layered understanding of vulnerability than a checklist approach can do.
